# The transcriptional regulator Fur modulates the expression of *uge*, a gene essential for the core lipopolysaccharide biosynthesis in *Klebsiella pneumoniae*

**DOI:** 10.1186/s12866-024-03418-x

**Published:** 2024-07-27

**Authors:** José Júlio Muner, Paloma Aparecida Alves de Oliveira, Juliana Baboghlian, Stefany Casarin Moura, Abissair Gabriel de Andrade, Michelly Macedo de Oliveira, Yasmin Ferreira de Campos, Alquiandra Stefani Ferreira Mançano, Nathália Maria Gonçalves Siqueira, Thaisy Pacheco, Lúcio Fábio Caldas Ferraz

**Affiliations:** 1 Laboratório de Microbiologia Molecular e Clínica, Universidade São Francisco, Bragança Paulista, SP Brazil; 2 Central Multiusuária de Análises Genômica e Transcriptômica (CmAGT), Universidade São Francisco, Bragança Paulista, SP Brazil

**Keywords:** *Klebsiella pneumoniae*, Lipopolysaccharides, Transcriptional regulation, Iron-regulatory proteins, Gene expression analyses

## Abstract

**Background:**

*Klebsiella pneumoniae* is a Gram-negative pathogen that has become a threat to public health worldwide due to the emergence of hypervirulent and multidrug-resistant strains. Cell-surface components, such as polysaccharide capsules, fimbriae, and lipopolysaccharides (LPS), are among the major virulence factors for *K. pneumoniae*. One of the genes involved in LPS biosynthesis is the *uge* gene, which encodes the uridine diphosphate galacturonate 4-epimerase enzyme. Although essential for the LPS formation in *K. pneumoniae*, little is known about the mechanisms that regulate the expression of *uge*. Ferric uptake regulator (Fur) is an iron-responsive transcription factor that modulates the expression of capsular and fimbrial genes, but its role in LPS expression has not yet been identified. This work aimed to investigate the role of the Fur regulator in the expression of the *K. pneumoniae uge* gene and to determine whether the production of LPS by *K. pneumoniae* is modulated by the iron levels available to the bacterium.

**Results:**

Using bioinformatic analyses, a Fur-binding site was identified on the promoter region of the *uge* gene; this binding site was validated experimentally through Fur Titration Assay (FURTA) and DNA Electrophoretic Mobility Shift Assay (EMSA) techniques. RT-qPCR analyses were used to evaluate the expression of *uge* according to the iron levels available to the bacterium. The iron-rich condition led to a down-regulation of *uge*, while the iron-restricted condition resulted in up-regulation. In addition, LPS was extracted and quantified on *K. pneumoniae* cells subjected to iron-replete and iron-limited conditions. The iron-limited condition increased the amount of LPS produced by *K. pneumoniae*. Finally, the expression levels of *uge* and the amount of the LPS were evaluated on a *K. pneumoniae* strain mutant for the *fur* gene. Compared to the wild-type, the strain with the *fur* gene knocked out presented a lower LPS amount and an unchanged expression of *uge*, regardless of the iron levels.

**Conclusions:**

Here, we show that iron deprivation led the *K. pneumoniae* cells to produce higher amount of LPS and that the Fur regulator modulates the expression of *uge*, a gene essential for LPS biosynthesis. Thus, our results indicate that iron availability modulates the LPS biosynthesis in *K. pneumoniae* through a Fur-dependent mechanism.

**Supplementary Information:**

The online version contains supplementary material available at 10.1186/s12866-024-03418-x.

## Background

*Klebsiella pneumoniae* is a Gram-negative pathogen responsible for a wide range of healthcare- and community-associated infections, mostly in immunocompromised patients [1, 2]. This bacterium has become a worldwide public health threat due to the emergence of hypervirulent and antibiotic-resistant strains causing various types of invasive infections [3–5]. In addition, the increasing number of severe community-acquired infections in healthy individuals has emphasized the importance of studying virulence mechanisms that determine the pathogenicity of *K. pneumoniae*.

Cell envelope components, such as polysaccharide capsules, siderophore-mediated iron acquisition, fimbrial adhesins, and LPS, are the main and best-studied virulence factors of *K. pneumoniae* [2, 6–8]. As the main structural component of the outer membrane (OM) of Gram-negative bacteria, the LPS provide a permeability barrier on the cell surface and contributes to the innate resistance of *K. pneumoniae* against antimicrobial compounds [9]. For instance, LPS are crucial components for the ability of pathogens to spread through the blood, causing sepsis and confer protection to *K. pneumoniae* against phagocytosis and the bactericidal action of human serum [7, 10–13].

LPS are composed of three structural domains: lipid A, the core oligosaccharide, and the O polysaccharide antigen [9]. The O1 antigen is the most common among *K. pneumoniae* clinical isolates and is associated with colonization and dissemination to internal organs, including the development of pyogenic liver abscesses [10, 14]. In *K. pneumoniae*, the biosynthesis of the O antigen is carried out by the *wb* gene cluster, while the *waa* (*rfa*) gene cluster is responsible for the core oligosaccharide biosynthesis [9, 10, 15, 16]. An essential gene for the biosynthesis of the LPS core oligosaccharide in *K. pneumoniae* is the *uge* gene, which encodes the uridine diphosphate (UDP) galacturonate 4-epimerase enzyme [16]. This enzyme belongs to the nucleotide sugar metabolism and converts uridine diphosphate glucuronic acid (UDP-GlnA) into uridine diphosphate galacturonic acid (UDP-GalA). GalA is the first outer core LPS residue on *K. pneumoniae* and presents a crucial role in the stability of the LPS structure [16, 17]. The absence of the *uge* gene makes the mutant *K. pneumoniae* completely avirulent in animal models of infections, and this was attributed to the presence of a truncated core oligosaccharide at the GalA residue [16], which reinforces the pivotal role of the *uge* gene in LPS biosynthesis pathways.

For most bacteria, iron is an essential element needed not only for growth and cellular metabolism but also as a cofactor for the regulation of many genes involved in bacteria virulence [18–21]. The transcriptional regulator Fur modulates the expression of genes related to iron homeostasis and pathogenicity in *K. pneumoniae* [22]. In its classical mode of action, Fur regulates gene expression by complexing with its cofactor, ferrous iron, and binding to regulatory sequences, called Fur boxes, located on the promoter region of the target genes [23].

In the present work, we describe the identification of a functional Fur box on the promoter region of the *uge*. Our data suggest that the expression of this gene is regulated by Fur and, consequently, that the production of LPS by *K. pneumoniae* can be modulated according to the levels of iron available to bacteria.

## Results

### The promoter region of *uge* harbors a functional DNA-binding site for regulator

**Fig. 1:**
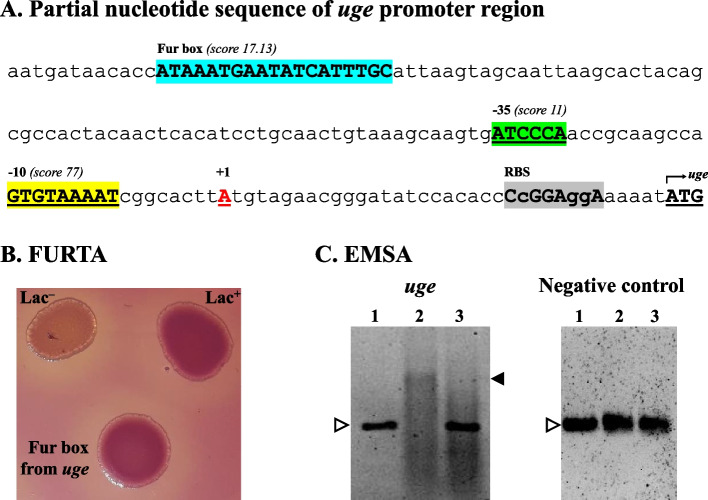
The promoter region of *uge* harbors a functional Fur binding box validated by FURTA and EMSA. **A** Partial nucleotide sequence of the 5’-upstream region of *uge* showing the initial codon (ATG, underlined) and the ribosome binding site (RBS, highlighted in grey). Uppercase nucleotides on RBS are identical to the *K. pneumoniae* RBS proposed by Kim et al. [24]. The transcription initiation site of *uge* is indicated at position + 1 (underlined Adenine in red). BPROM program predicted the -35 (in green) and -10 (in yellow) domains of the Sigma factor RpoD. The putative Fur binding box, identified 154 nucleotides upstream of the *uge* start codon, is highlighted in blue. The functionality of this Fur box was experimentally validated by FURTA and EMSA. **B** FURTA, *E. coli* H1717 transformed with a functional Fur box will appear red on MacConkey agar plates (positive control, Lac^+^ phenotype), whereas H1717 transformed with a non-functional Fur box will appear colorless (negative control, Lac^–^ phenotype). FURTA validated the Fur box from *uge*, as indicated by the red colonies of *E. coli* H1717 transformed with the Fur box sequence from *uge* (FURTA-positive phenotype). **C** EMSA, a mobility shift was observed when the DNA probes containing the Fur box from *uge* were incubated with *K. pneumoniae* purified His-Fur protein in the presence of divalent cation (lane 2), compared to the DNA probes alone (without the addition of His-Fur protein, lane 1). The mobility shift of the probes was abolished under divalent cation-free conditions (in the presence of the chelator EDTA, lane 3). No mobility shift was observed with the DNA probe without the Fur box sequence from *uge* (the negative control). Open arrowhead indicates the DNA probes alone, while closed arrowhead indicates the mobility shift corresponding to the Fur/DNA complexes. Full-length gels are presented in Supplementary Figure S1

Bioinformatic analyses were performed to identify regulatory elements on the promoter region of the *uge* gene from *K. pneumoniae*. As shown in Table 1, a putative Fur binding box was identified 154 bases upstream from the ATG start codon of the *uge* gene. Figure 1A shows the location of the Fur box on the promoter region of *uge*, along with other regulatory elements such as Sigma factor domains, the transcription initiation site and the ribosome binding site (RBS). The RBS and the transcription initiation site of *uge* were determined according to Kim et al. [24], while BPROM program predicted the -35 and -10 domains of the Sigma factor RpoD. The putative Fur box was experimentally validated by Fur Titration Assay (FURTA, Fig. 1B) and Electrophoretic Mobility Shift Assay (EMSA, Fig. 1C).

**Table 1:** The putative Fur box identified on the promoter region of the *uge* gene

Position^1^	Fur box sequence^2^	Score^3^
154 bp	ATAAATGAATATCATTTGC	17.13

^1^Location in base pairs (bp) upstream of the *uge* start codon

^2^Underlined nucleotides are identical to the proposed Fur-consensus sequence [23]

^3^Score expressed in bits

FURTA revealed that Fur regulator from *E. coli* H1717 was able to bind to the putative Fur box from *uge* of *K. pneumoniae*, as revealed by H1717 red colonies on MacConkey plates (Lac^+^, FURTA-positive phenotype, Fig. 1B). EMSA confirmed the direct interaction of the *K. pneumoniae* His-Fur protein on the Fur box identified on *uge*. Fur interaction requires divalent cations since mobility shift was not observed under divalent cation-free conditions (Fig. 1C). No mobility shift was observed in the EMSA with the DNA probe without the Fur box sequence (negative control).

### The expression of *uge* and the LPS production is modulated according to the availability of **iron** in the culture medium

**Fig. 2:**
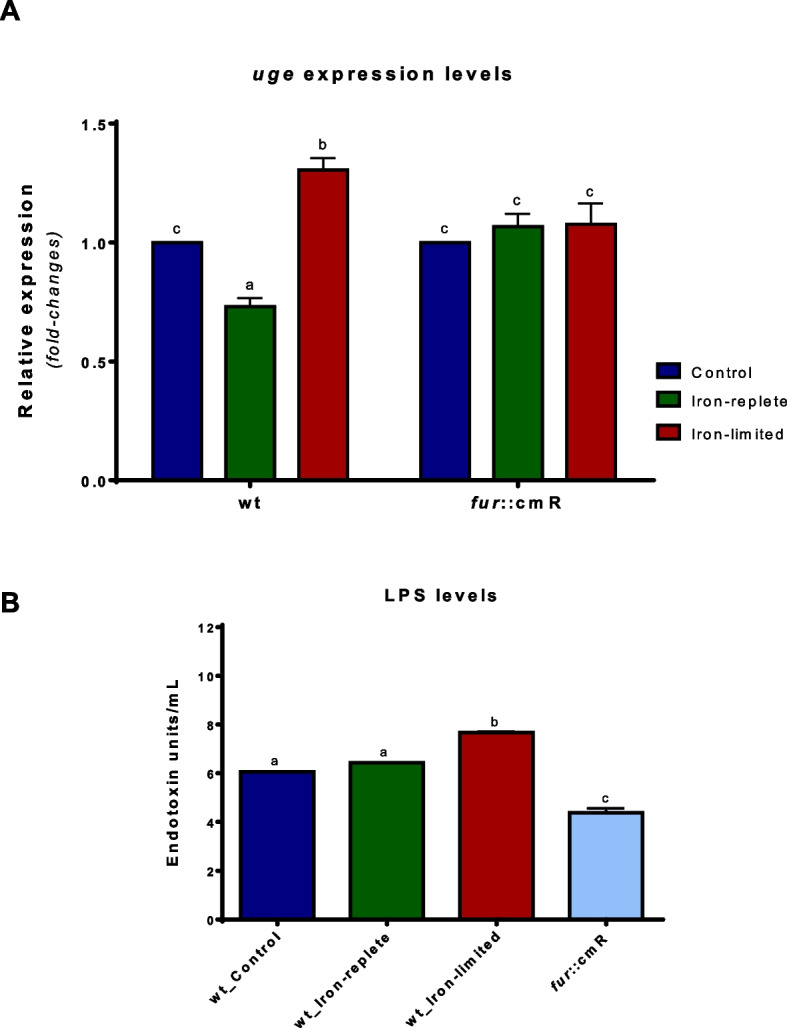
Iron levels modulate the expression of *uge* and the amounts of LPS produced by *K. pneumoniae*. **A** RT-qPCR analyses showed down-regulation and up-regulation of *uge* expression, respectively, under iron-replete (FeSO4) and iron-limiting (dipyridyl) conditions in the ATCC 10031 wild-type (wt) strain. The strain mutant for the *fur* gene has unchanged expressions of *uge* in all the conditions tested. **B** Iron-limited conditions led the *K. pneumoniae* cells to produce higher amounts of LPS when compared to the control and iron-replete (FeSO4) conditions. The iron-rich condition presented no change in LPS production compared to the control condition. The *fur* mutant strain produces less LPS than the ATCC 10031 strain, regardless of the culture conditions in which the strain is submitted. Different letters above the graphical bars indicate statistically significant differences ( *p*-value < 0.05)

The presence of a functional Fur box on the *uge* promoter region prompted us to analyze how the iron available to the *K. pneumoniae* cells modulates the expression of *uge*. When compared to the control condition, the expression of *uge* was slightly down-regulated under iron-replete condition and up-regulated under iron-limiting condition in the wild-type strain ATCC 10031 (Fig. 2A). To investigate whether the modulation of *uge* expression according to iron levels is indeed modulated by Fur regulator, *uge* expression was also assessed in a *K. pneumoniae* strain mutant for the *fur* gene. As shown on Fig. 2A, the *fur* mutant strain has unchanged expressions of *uge* under all tested conditions.

To further understand the effects of iron availability on LPS biosynthesis, the levels of LPS were quantified on *K. pneumoniae* cells subjected to iron-replete and iron-limited conditions. As displayed on Fig. 2B, no change in LPS quantification was observed when the iron-replete and the control conditions were compared. However, the iron starvation led to the production of higher amounts of LPS compared to the control and iron-replete conditions. Figure 2B also shows that the *fur* mutant strain produces LPS at a lower rate than the ATCC 10031 wild-type (wt) strain, regardless of the iron levels.

## Discussion

Cell-surface components, such as polysaccharide capsules, fimbrial adhesins and LPS, are among the virulence factors that contribute to *K. pneumoniae* pathogenicity [2, 25]. Previous reports have demonstrated the role of Fur regulator on the expression modulation of genes involved in the biosynthesis of fimbriae [22, 26], polysaccharide capsule [27, 28], and siderophore receptors for iron uptake [29], according to the iron levels available to the bacteria. Here, we report evidence suggesting that iron availability also modulates the LPS biosynthesis in *K. pneumoniae* through a Fur-dependent mechanism.

In this study, we focused on the mechanisms that regulate the expression of the *uge* gene which encodes an enzyme that converts UDP-GlcA to UDP-GalA, the first outer core LPS residue in *K. pneumoniae* and confers stability to the structure [16, 17]. The importance of the *uge* gene on the core LPS biosynthesis and virulence of *K. pneumoniae* was demonstrated by Regué and colleagues [16]. These authors observed that the loss of *uge* leads to mutant *K. pneumoniae* cells with a truncated LPS core without O antigen molecules and outer core oligosaccharides in its structure. Furthermore, those authors found that the mutant strain was unable to colonize the urinary tract in rats and was avirulent in murine septicemia and pneumonia models [16].

First, we described the identification of a functional Fur regulator binding site on the *uge* promoter region, validated by two independent techniques: FURTA and EMSA. We also showed that Fur regulates the transcription of *uge* depending on the iron levels. We observed that Fur complexed with ferrous iron binds on the promoter region of *uge* and down-regulates its expression. In the absence of iron, Fur no longer binds to *uge* promoter and the gene is up-regulated. This mechanism of action resembles the traditional mode of action of the Fur regulator. In this classic mechanism, Fur functions as a transcriptional repressor that forms a complex with its corepressor, ferrous iron, and this complex binds to the promoter region of target genes, causing their repression by preventing the binding of RNA polymerase to the genes’ promoter regions. Interestingly, while Fur indirectly controls the expression of capsular and fimbrial genes by modulating the expression of transcriptional regulators of fimbriae [22, 26] and capsules [27, 28], here we show that Fur participates in LPS biosynthesis by directly regulating the expression of *uge*, a gene whose product is essential for the production of LPS oligosaccharides.

We also observed that the availability of iron slightly modulates the LPS production in *K. pneumoniae*. No change in LPS production was observed on *K. pneumoniae* cells grown under iron-replete condition. However, the iron deprivation led the cells to produce a higher amount of LPS compared to the control and iron-replete conditions. The role of iron on LPS production by Gram-negative is still elusive and poorly understood. On the other hand, the effects of iron availability on the chemical composition of LPS are well-documented. For instance, Keenan and colleagues [30] showed that iron-limited conditions affect the extent and nature of the LPS O antigen chain in *Helicobacter pylori*. Strikingly, while here we report an increase in the LPS amounts by *K. pneumoniae* cells subjected to iron-limited conditions, those authors described shorter LPS in *H. pylori* cells subjected to the same conditions. According to the authors, *H. pylori* grown in iron-limited conditions has reduced levels of ATP, which impairs the translocation of the core-lipid A domain across the inner membrane and the final step in LPS synthesis on the outer membrane [30]. Moreover, Keenan and coauthors found that the influence of iron on *H. pylori* LPS takes place on the synthesis of the O antigen chain [30], whereas we show that iron starvation up-regulates the expression of the *uge* gene, whose product is involved in the biosynthesis of core oligosaccharides of *K. pneumoniae* LPS, and leads the bacteria to produce a higher amount of LPS. Furthermore, in numerous Gram-negative bacteria [31], including *K. pneumoniae* [32], extracellular iron activates the two-component system PmrA/PmrB, which regulates the expression of genes involved in the chemical modifications of LPS with profound consequences for antibiotic resistance [33–36]. In the PmrA/PmrB system, environmental stimuli such as high extracellular iron, are detected by the sensor tyrosine kinase protein PmrB, which phosphorylates the regulatory protein PmrA. Phosphorylated PmrA induces the expression of target genes by binding to their promoter region [35–38]. The PmrA-regulated genes encode proteins that introduce modifications into the LPS, such as the addition of phosphoethanolamine to lipid A, rendering the LPS less negatively charged and promoting resistance to the cationic peptide antibiotic polymyxin [35–37].

LPS are essential for the stability of the bacterial cell envelope, serving as a permeability barrier and in the resistance against antimicrobials [9]. Therefore, LPS levels must be under tight control to ensure proper balance: too much LPS is toxic and lethal, while too little trigger cell envelope stress that leads to cell death [39]. Here, we show that the changes in the LPS and the *uge* gene expression levels are slightly modulated by the iron availability in a Fur-dependent manner. Although originally described as a transcriptional regulator of genes related to iron homeostasis, Fur is recognized as a global regulator that modulates the expression not only of genes involved in iron metabolism but also in many aspects of bacterial physiology, including pathogenicity [40, 41]. Thus, the slight differences in the expression of *uge* seem to emphasize that LPS biosynthesis may not rely on only one exclusive and global transcriptional regulator. We may infer that other regulators may presumably be acting in conjunction with the Fur regulator in the expression modulation of this gene, resulting in a tight and finely-tuned regulation. Indeed, given the complex composition of LPS and their role in the bacterial envelope, its biosynthesis certainly involves other regulators to achieve a more complex and intricate regulatory network [39, 42].

Our study presents some limitations. For instance, we did not investigate the influence of iron on the expression regulation of the *wb* and *waa* LPS biosynthesis gene clusters. In our study, we observed an enhanced production of LPS by *K. pneumoniae* under iron deficiency, but we also did not perform in-depth analyses to determine the chemical composition of the LPS core in *K. pneumoniae* cells under iron scarcity, a condition in which the *uge* gene is up-regulated. Since the enzyme encoded by *uge* is responsible for GalA synthesis and this sugar acid is present in the *K. pneumoniae* core LPS, we can merely hypothesize that the bacterium under iron-scarce conditions would have increased amounts of GalA among the oligosaccharides on the LPS core backbone. Furthermore, it cannot be excluded that the slight difference in the LPS production may be due to the concentrations of iron chelator and iron salts used in the present study. For further studies, assessment of the LPS production in bacteria cultured under higher concentrations of iron and iron chelator, LPS chemical analysis by mass spectrometry on bacteria subjected to different levels of iron, and a more comprehensive analysis of the expression of LPS biosynthesis genes according to available iron levels are suggested to fully understanding the specific alterations induced by iron availability.

## Conclusions

In summary, here we show that iron deprivation increases the amount of LPS produced by *K. pneumoniae* and that the Fur regulator modulates this effect by regulating the expression of *uge*, a gene involved in sugar metabolism and essential for the synthesis of the core oligosaccharide of LPS. The present study represents an important step toward increasing our understanding of LPS biosynthesis in the context of iron availability. Our study serves as a starting point for future research on a relevant and poorly addressed issue that needs to be better explored: the effects of iron on LPS biosynthesis and the potential implications of these effects on the virulence of Gram-negative bacteria.

## Methods

### Bacterial strains and culture conditions

This study was conducted with a wild-type (ATCC 10031, USA) and a *fur* mutant *Klebsiella Klebsiella pneumoniae* strains. The *fur* mutant strain was acquired from the Laboratory of Dr. Colin Manoil, University of Washington, USA [43, 44]. The gene knockout was obtained by inserting a transposon harboring a gene conferring resistance to the antibiotic chloramphenicol (cm^R^) inside the coding region of the *fur* gene; for this reason, the *fur* mutant strain was named *fur*::cm^R^. Sanger sequencing performed on the C_m_AGT lab confirmed the disruption of the *fur* gene. The bacterial strains were cultured in LB (Luria-Bertani) broth Miller (BD Biosciences, USA) at 37 °C either with shaking at 200 rpm or on LB agar under static conditions. Bacterial growth was monitored by measuring the optical density (O.D.) of the cultures at a wavelength of 600 nanometers (O.D._600nm_) using the GeneQuant Spectrophotometer (GE Healthcare, USA). Growth under iron-replete and iron-limiting conditions were obtained by supplementing the LB broth with a source of ferrous iron (FeSO_4_, Sigma-Aldrich, India) or with the iron chelator 2,2’-dipyridyl (Sigma-Aldrich, China) to a final concentration of 100 μM [22, 29].

### Identification and validation of putative **Fur** boxes on *uge* gene promoter

Putative Fur-binding sites were screened on the promoter region of *uge* by using a theoretical approach previously adapted to *K. pneumoniae* [29, 45]*.* Prediction of possible Sigma factors sequence motifs was performed with the web-based program *BPROM-Prediction of Bacterial Promoters*, as previously described [22, 46]. This predictive analysis was performed on 200 nucleotides upstream to the initial codon of *uge* gene. The predicted ribosome binding site (RBS) and the transcription start site (TSS) of *uge* were identified according to Kim and coauthors [24].

The functionality of the putative Fur box identified on the upstream region of *uge* was independently validated by two assays, Fur Titration Assay (FURTA) and DNA Electrophoretic Mobility Shift Assay (EMSA), following protocols previously described by our team [22, 29]. In brief, the double-stranded sequence of the putative Fur box was cloned into pGEM^®^-T Easy vector (Promega, USA) and used in FURTA and EMSA. For FURTA, the reporter strain *Escherichia coli* H1717 (kindly provided by Prof. Klaus Hantke, University of Tübingen, Germany) was transformed with the recombinant vector and plated on agar MacConkey (Kasvi, Spain). If transformed with a vector cloned with a functional Fur box, H1717 colonies will appear red on MacConkey agar plates (Lac^+^ phenotype). Otherwise, H1717 colonies will appear colorless if transformed with a vector cloned with a non-functional Fur box. For negative control, *E. coli* H1717 were transformed with recircularized pGEM^®^-T Easy with no insert. For positive control, H1717 was transformed with a vector cloned with a previously validated Fur box of *K. pneumoniae* [29].

EMSA was performed with 50 nanograms of the DNA probes and 500 nM of *K. pneumoniae* purified Histidine-tagged recombinant Fur protein, following protocols previously reported by our team [29, 47]. Briefly, the entire coding region of the *fur* gene was PCR amplified from the *K. pneumoniae* DNA chromosome and the amplicon was inserted into the expression vector pET-28a (Novagen, USA). The recombinant vector was introduced into *E. coli* BL21(DE3) (Novagen, USA), and the Histidine-tagged recombinant Fur protein (His-Fur) was overexpressed with isopropyl-β-D-thiogalactopyranoside (IPTG, Invitrogen™, Italy). After IPTG induction, in-culture bacterial cell lysis was promoted by adding CelLytic™ Express 1 mL Tablets (Sigma-Aldrich, USA), as recommended by the manufacturer. The lysed cells were centrifuged at 16,000 *g* for 15 min to obtain a clarified supernatant. The His-Fur was purified from the clarified supernatant by affinity chromatography under native conditions using Ni–NTA Agarose Matrix (Qiagen, Germany), following the instructions of the manufacturer. Fractions of the His-Fur protein eluted from the Ni–NTA Agarose were pooled and dialyzed overnight at 4 °C on storage buffer (20 mM Tris–HCl pH 7.8, 1 mM DTT, 0.1 mM MnSO4 and 10% glycerol v/v). The purified His-Fur proteins were concentrated using the Pierce™ Protein Concentrators (Thermo Scientific™, United Kingdom) with molecular weight cutoff of 10 kDa, according to the manufacturer’s protocol. The concentration of the purified His-Fur was determined by the Bradford method (Bradford Reagent, Sigma-Aldrich, USA) and the purity was verified by SDS-PAGE analysis.

The DNA probes were obtained by PCR amplifying the vector cloned with the putative Fur identified on *uge* gene using the universal M13-Forward (5’-GTTTTCCCAGTCACGAC-3’) and M13-Reverse (5’-CAGGAAACAGCTATGAC-3’) primers, with the annealing temperature of 55 °C. The DNA probe for the negative control consisted of amplicons obtained from recircularized vector with no insert. Since Fur may require divalent ions for binding on DNA, the binding reactions were performed under the presence and absence of divalent cations. To supply the divalent cations, MgCl_2_ and MnSO_4_ (Sigma-Aldrich, Japan) were added to the reaction to a final concentration of 0.5 mM each. For binding reactions under divalent cation-free conditions, the chelating agent EDTA (Sigma-Aldrich, USA) was added to a final concentration of 2 mM. After incubation on ice for 20 min on the proper binding buffer, the mixtures were subjected to electrophoresis on a 2% (w/v) agarose gel (Ludwig Biotec, Brazil) prepared with 1 × Sodium Borate buffer (Synth, Brazil) containing 0.1 mM MnSO_4_. Then, the gels were stained with 0.5 µg/ml of ethidium bromide solution (Sigma-Aldrich, USA) and the protein-DNA complexes were visualized under UV light using the Molecular Imager^®^ Gel Doc™ XR System (Biorad, USA) with the Image Lab™ Software version 5.0 (Biorad, USA).

### RNA extraction and Real-Time Quantitative PCR Analysis

The transcript levels of *uge* were evaluated in wild-type (ATCC 10031) and *fur* mutant strains subjected to iron-replete and iron-limited conditions. These analyses were performed using Reverse Transcription Quantitative Real-Time Polymerase Chain Reaction (RT-qPCR). Initially, *K. pneumoniae* cells were grown in LB broth at 37 °C under agitation until saturation (overnight). The next day, the culture was diluted with LB broth to O.D._600nm_ of 1.0, and this suspension was used to inoculate fresh LB broth with a proportion of 1:200 (bacterial suspension: LB broth). Inoculations were done on three conditions: LB broth supplemented with FeSO_4_ (for iron-replete condition), with the iron chelator 2,2’-dipyridyl (for iron-restricted condition) or with no supplements (for control condition). The inocula were cultured at 37 °C with shaking until they reached the initial logarithmic phase of growth (O.D._600nm_ of 0.4). Supplementary Figure S2 shows the growth patterns of the strains under the tested conditions. At this point, the bacterial cells were harvested by centrifugation, and the pellets were resuspended in RNAprotect^®^ Bacteria Reagent (Qiagen, Germany) for RNA stabilization and immediately submitted to total RNA extraction.

Total bacterial RNA and cDNA synthesis were performed following protocols published elsewhere [22, 47]. RT-qPCR reactions were performed in triplicates on the 7300 Real-Time PCR System (Applied Biosystems™, Singapore) with the Platinum™ SYBR™ Green qPCR SuperMix-UDF w/Rox kit (Invitrogen™, USA), according to the manufacturer’s instruction and following a protocol previously reported [47]. RT-qPCR results were normalized using *rho* as an endogenous control gene [29], and the relative expression levels were calculated using the 2^−ΔΔCt^ relative quantification method [48]. Primers used on the RT-qPCR reactions are displayed in Table 2 and were designed using Primer3web version 4.1.0 [49, 50] in order to present 60 °C of annealing temperature and to render amplicon sizes ranging from 95 to 105 base pairs (bp). RT-qPCR analyses were performed from each culture condition performed at least thrice.

**Table 2:** Primer forward (F) and reverse (R) used on RT-qPCR reactions

Genes	*Primers*	Sequence (5’-3’)	Amplicon
*uge*	*uge*-F	ATGGGGTATCTGAACATTCTGG	104 bp
	*uge*-R	AACGGCATTTTACGGTTAAGG	
*rho*^1^	*rho*-F	AACTACGACAAGCCGGAAAA	99 bp
	*rho*-R	ACCGTTACCACGCTCCATAC	

^1^Endogenous control gene used to normalize the RT-qPCR reactions [29]

### Endotoxin lipopolysaccharides (LPS) extraction and quantification

LPS were extracted and quantified from *K. pneumoniae* ATCC 10031 cells subjected to iron-replete and iron-limited conditions, in order to investigate the effects of iron levels on LPS production. Saturated cultures of *K. pneumoniae* were diluted with LB broth to O.D._600nm_ of 1.0 and used to inoculate fresh LB broth supplemented with FeSO_4_, 2,2’-dipyridyl or with no supplements in a proportion of 1:200. Bacteria were cultured at 37 °C with shaking until they reached the O.D._600nm_ of 0.4, at the initial logarithmic growth phase. At this point, the bacterial cells were harvested by centrifugation, the supernatants were discarded, and the cell pellets were submitted to the LPS extraction and purification using the Lipopolysaccharide (LPS) Isolation Kit (MAK339, Sigma-Aldrich, USA), following the instructions of the manufacturer with minor modifications. In brief, 10 μL of lysis buffer provided by the kit were added per milligram wet weight of the pellets. Samples were sonicated and kept on ice to complete lysis and then were treated with proteinase K and nucleases to remove contaminating protein and nucleic acids. The endotoxin LPS levels were determined using the Pierce™ Chromogenic Endotoxin Quant Kit (Thermo Scientific™, USA), according to the manufacturer's protocol. The absorbance of the reaction product was measured at 405 nanometers in a microplate reader (Biochrom Asys, United Kingdom), and the endotoxin concentrations were calculated using the “low” standard curve provided by the kit. According to the protocol, the results are expressed as Endotoxin Units per milliliter (EU/mL). LPS was quantified from each culture condition performed at least thrice.

### Statistical analysis

GraphPad Prism 7.0 (GraphPad Software, Inc., USA) was used for the statistical analyses. Comparisons of the LPS production and the *uge* expression levels among the experimental groups were conducted using two-way ANOVA with Tukey’s multiple comparisons test. Differences were considered statistically significant at *p*-value < 0.05.

### Supplementary Information

Supplementary Material 1.

## Data Availability

The datasets generated and analyzed during the current study are available from the corresponding author upon reasonable request.
