# Supplementary material for: The transcriptional regulator Fur modulates the expression of uge, a gene essential for the core lipopolysaccharide biosynthesis in Klebsiella pneumoniae
Source: BMC Microbiol. 2024 Jul 27;24:279. doi: 10.1186/s12866-024-03418-x (PMC11282780; doi:10.1186/s12866-024-03418-x)
Supplement: Supplementary file 1 — Supplementary Material 1. [file 12866_2024_3418_MOESM1_ESM.pdf]

SUPPLEMENTARY INFORMATION

The transcriptional regulator Fur modulates the expression of *uge*, a gene essential for the core lipopolysaccharide biosynthesis in *Klebsiella pneumoniae*

José Júlio Muner[†,1], Paloma Aparecida Alves de Oliveira[†1,2], Juliana Baboghlian[†1], Stefany Casarin Moura[1], Abissair Gabriel de Andrade[1], Michelly Macedo de Oliveira[1], Yasmin Ferreira Campos[1], Alquiandra Stefani Ferreira Mançano[1], Nathália Maria Gonçalves Siqueira[1], Thaisy Pacheco, Lúcio Fábio Caldas Ferraz[*1,2]

[1]Laboratório de Microbiologia Molecular e Clínica, Universidade São Francisco, Bragança Paulista, SP, Brazil

[2]Central multiusuária de Análises Genômica e Transcriptômica (C_mAGT), Universidade São Francisco, Bragança Paulista, SP, Brazil

*** Correspondence:**
Lúcio Fábio Caldas Ferraz
lucio.ferraz@usf.edu.br

[†]These authors contributed equally to this work and should be considered the first authors.

SUPPLEMENTARY FIGURES

a)

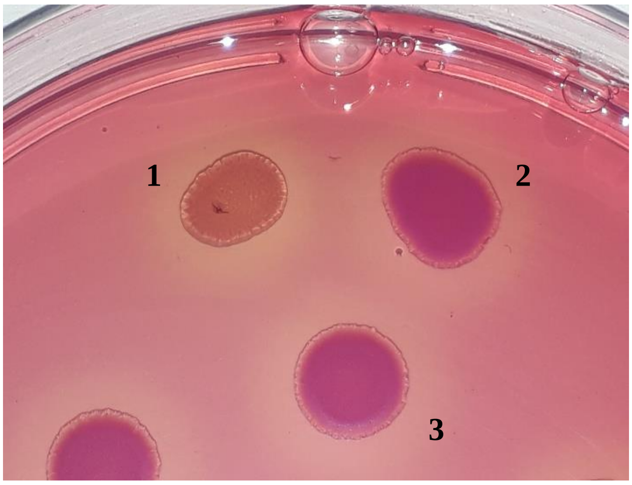

1. Furta-negative phenotype (Lac $^{-}$).
2. Furta-positive phenotype (Lac $^{+}$).
3. Furta with the Fur box from *uge*.

b)

uge **Negative control**

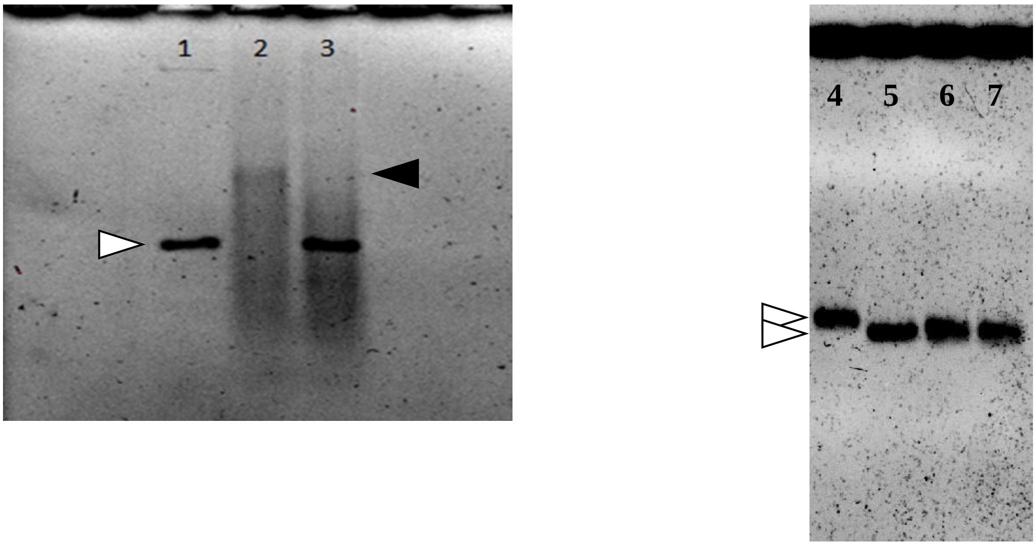

FIGURE S1. Full-length figures showing the validation of the putative Fur box identified on the promoter region of *uge* gene by FURTA (**a**) and EMSA (**b**).

a) **FURTA:** Lac^{+} indicates Furta-positive phenotype, whereas Lac^{-} indicates Furta-negative phenotype. The putative Fur box on *uge* resulted on red *E. coli* H1717 colonies on MacConkey plates, which is interpreted as a Furta-positive result.

b) EMSA: EMSA were performed with 500 ηM of *K. pneumoniae* purified His-tagged recombinant Fur protein and 50 ηg of the DNA probes. The DNA probes containing the Fur box from *uge* were obtained by PCR amplifying the vector pGEM®-T Easy cloned with the Fur box sequence using the universal M13 primers, which renders an amplicon of 285 base pairs. The DNA probe without Fur box sequence (the negative control) consisted of a 254 base pairs DNA fragment obtained by PCR amplifying the recircularized vector with no insert.

Lanes 1, 2, 3 and 4 contain the DNA probes harboring the Fur box from *uge*, while lanes 5, 6 and 7 contain DNA probes without Fur box (the negative control). Lanes 1, 4 and 5 show the probes alone (without addition of the Fur protein). On lanes 2 and 6, the probes were incubated with Fur protein in the presence of divalent cation. On lanes 3 and 7, the probes were incubated with Fur protein under divalent cation-free conditions by adding 2 mM EDTA. Open arrowheads indicate the free DNA probes, while closed arrowheads indicate the mobility shift corresponding to the Fur/DNA complexes. No mobility shift was observed with the DNA probes without the Fur box sequence from *uge* (the negative control).

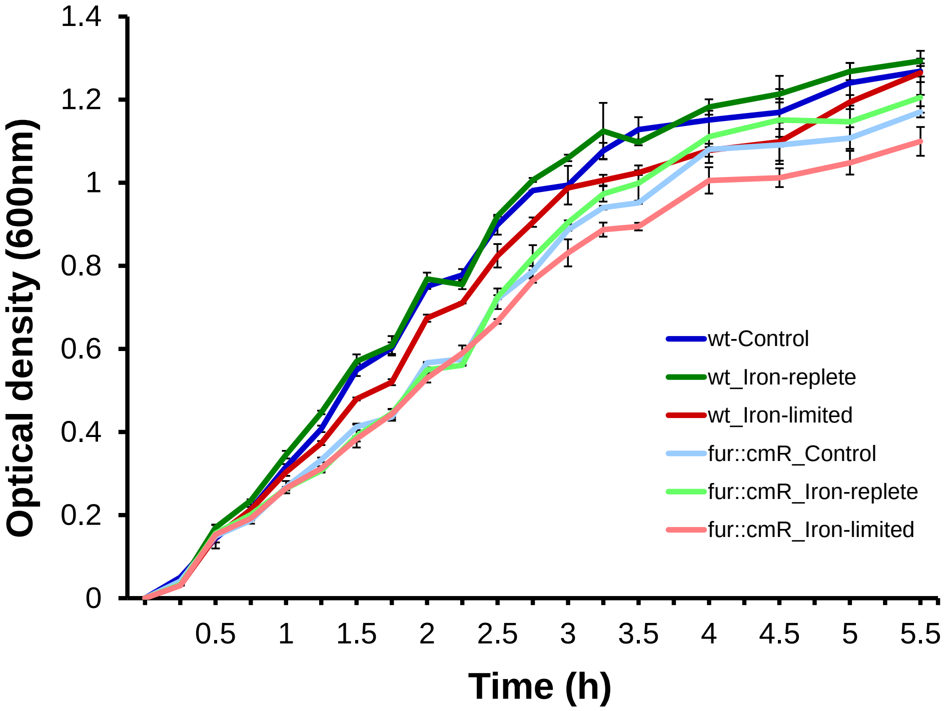

Figure S2. Growth curves of the wild-type (wt) and the *fur* mutant (*fur*::cm^{R}) strains of *K. pneumoniae* subjected to the control, iron-replete, and iron-limited conditions. The RT-qPCR analyses and the LPS extraction and quantification assays were performed with cells harvested at the beginning of the exponential, or logarithmic, phase of growth (O.D.$_{600nm}$ of 0.4). Data from each point of the curves are displayed as the mean and standard deviation of three independent experiments for each indicated time.
